# Going Remote—Demonstration and Evaluation of Remote Technology Delivery and Usability Assessment With Older Adults: Survey Study

**DOI:** 10.2196/26702

**Published:** 2021-03-04

**Authors:** Jordan R Hill, Addison B Harrington, Philip Adeoye, Noll L Campbell, Richard J Holden

**Affiliations:** 1 Department of Medicine Indiana University School of Medicine Indianapolis, IN United States; 2 Center for Aging Research Regenstrief Institute Indianapolis, IN United States; 3 Department of Pharmacy Practice College of Pharmacy Purdue University West Lafayette, IN United States

**Keywords:** COVID-19, mobile usability testing, usability inspection, methods, aging, agile, mobile phone

## Abstract

**Background:**

The COVID-19 pandemic necessitated “going remote” with the delivery, support, and assessment of a study intervention targeting older adults enrolled in a clinical trial. While remotely delivering and assessing technology is not new, there are few methods available in the literature that are proven to be effective with diverse populations, and none for older adults specifically. Older adults comprise a diverse population, including in terms of their experience with and access to technology, making this a challenging endeavor.

**Objective:**

Our objective was to remotely deliver and conduct usability testing for a mobile health (mHealth) technology intervention for older adult participants enrolled in a clinical trial of the technology. This paper describes the methodology used, its successes, and its limitations.

**Methods:**

We developed a conceptual model for remote operations, called the Framework for Agile and Remote Operations (FAR Ops), that combined the general requirements for spaceflight operations with Agile project management processes to quickly respond to this challenge. Using this framework, we iteratively created *care packages* that differed in their contents based on participant needs and were sent to study participants to deliver the study intervention—a medication management app—and assess its usability. Usability data were collected using the System Usability Scale (SUS) and a novel usability questionnaire developed to collect more in-depth data.

**Results:**

In the first 6 months of the project, we successfully delivered 21 *care packages*. We successfully designed and deployed a minimum viable product in less than 6 weeks, generally maintained a 2-week sprint cycle, and achieved a 40% to 50% return rate for both usability assessment instruments. We hypothesize that lack of engagement due to the pandemic and our use of asynchronous communication channels contributed to the return rate of usability assessments being lower than desired. We also provide general recommendations for performing remote usability testing with diverse populations based on the results of our work, including implementing screen sharing capabilities when possible, and determining participant preference for phone or email communications.

**Conclusions:**

The FAR Ops model allowed our team to adopt remote operations for our mHealth trial in response to interruptions from the COVID-19 pandemic. This approach can be useful for other research or practice-based projects under similar circumstances or to improve efficiency, cost, effectiveness, and participant diversity in general. In addition to offering a replicable approach, this paper tells the often-untold story of practical challenges faced by mHealth projects and practical strategies used to address them.

**Trial Registration:**

ClinicalTrials.gov NCT04121858; https://clinicaltrials.gov/ct2/show/NCT04121858

## Introduction

### Overview

Technological advances have made it more commonplace and easier for individuals to complete tasks remotely, from online banking to visiting a doctor to paid work from home [1]. The COVID-19 pandemic revealed that remote interaction is also sometimes necessary. The need to “go remote” during the pandemic likely both accelerated innovation in telecommunication and normalized available but underused internet-based services and products, such as telehealth [2], mental health apps [3], and e-commerce [4].

The pandemic also disrupted research, forcing projects to suspend operations or implement remote noncontact methods [5-7]. Paradoxically, some studies of mobile technology faced difficulties converting to entirely remote technology delivery and usability assessment, revealing opportunities for technology projects to experiment with remote operations [8].

This paper reports our experiences in “going remote” to rapidly restart a suspended clinical trial of a mobile health (mHealth) app with older adults. We present our protocol changes, Agile project management approach, and findings from this restart project. Our experiences elucidate the opportunities and challenges of conducting technology research remotely and may inform other attempts at rapid operational change.

### Background

Remote delivery and usability assessment of technology is not new [9,10] but have heretofore been largely used with younger, highly educated, technologically experienced, and more homogeneous individuals, for example, college students [9,11] or those using the technology as a part of their employment [12-14]. Only one study was found to employ remote methods with older adults and these were supplemented with in-person sessions for initial training and instruction [15].

Nevertheless, existing remote methods provide a foundation for technology development projects with older, disabled, or other diverse populations in which experience is limited. Potentially useful existing methods to access and evaluate technology include online questionnaires, online forums, self-kept diaries, email, telephone calls, and postal delivery [11,15-17]. More recently, videoconferencing tools are used to replicate in-person, moderated usability testing [8,11,17].

These methods have, to our knowledge, not been adapted for fully remote projects with older adult users, although some work has been done with younger and older adults with various disabilities [18]. This gap is relevant because older adults are the fastest growing population of technology users [19] but also one of the most diverse populations in terms of their motivations to use technology and their physical, cognitive, and sensory capabilities [20]. Additionally, among older adults, factors such as race and ethnicity, income, literacy, education level, age group (eg, <75 vs ≥75 years of age), and community characteristics (eg, rural vs urban) significantly impact access and use of the internet and other technologies [21-26]. Thus, one challenge of “going remote” with older adults is to accommodate between-age and within-age diversity.

Accordingly, prior research has suggested best practices and adjustments for in-person technology assessment with older adults. For example, because think-aloud procedures and psychometric scales (eg, Likert) may not work well for this population, it may be better to engage older adults one-on-one in a guided interview to elicit information [27,28]. Others recommend personalizing usability testing protocols with attention to fidelity in those with cognitive decline or disabilities [29].

### Objective

The objective of the reported project, Project C (COVID), was *to remotely deliver and conduct usability testing for an mHealth technology intervention for older adult participants enrolled in a clinical trial of the technology*. The need for remote delivery arose from restrictions on in-person research activities due to a global pandemic and an ethical obligation to reduce risk for older adult participants and study personnel. Meeting the objective required special effort due to participants’ diverse technology ownership, experience, motivations, and skill levels. For example, a subset of participants did not have a smartphone or home internet and required the study to provide these. Thus, the team could not simply provide an online link to the app for all participants to download. Below, we describe how we developed these remote operations to deliver and test mHealth technology to a heterogenous older adult population participating in human subjects research during a pandemic.

## Methods

### Overview

Project C occurred in the context of the Brain Safe randomized clinical trial, described in depth elsewhere [30]. The study enrolled adults aged 60 years or older in Indiana, USA, who were using medications with anticholinergic effects that may increase risk to brain health for older adults. In the trial’s parallel treatment arms, participants received a mobile app to use for 12 months, either the Brain Safe intervention app or the Med Safe attention control app. Both were native Android or iOS apps, Health Insurance Portability and Accountability Act (HIPAA) compliant, and optimized for smartphones, with the following features:

1. Self-registration.
2. Passwordless log-in, via verification token for each log-in.
3. Four-tab navigation.
4. A medication list created by the user through searching and browsing, selecting medication choices, and numeric data entry.
5. Monthly reminders to complete medication review.
6. Text and video educational materials.
7. PDF report output and sharing.
8. A score calculator.

Whereas the score calculator in the Med Safe app merely calculated the total number of medications, the Brain Safe app calculated a brain harm risk score, let users simulate the effect on risk of adding or removing anticholinergic medications, showed alternative treatments, and helped users start a conversation with their health care professionals about medication safety. Additional detail on features, design, development, and prior testing are available elsewhere [30,31].

Both apps were designed to be loaded on a smartphone meeting minimum hardware, software, and connectivity requirements. Originally, standard operating procedures in the trial were to conduct installation, account registration, training, first-time use, initial technical support, troubleshooting, and usability observations in person at the participant’s home or in a research office, with continuing opportunities for remote or in-person support. A smartphone, typically a 5.8-inch Samsung Galaxy S9 or S10, with voice calling and unlimited text and data plans was provided to any participant needing it for the study’s duration.

Project C was launched on March 13, 2020, when in-person trial operations were suspended due to COVID-19-related institutional policies, city and state governmental mandates, and an ethical imperative to reduce risk to participants, study personnel, and the community. At the time, the study had consented and enrolled 56 participants out of a planned 700. Of those enrolled, 7 (13%) had received the Brain Safe or Med Safe app and the remaining 49 (88%) were the target group for which Project C was designed. Over time, Project C provided remote capability for newly enrolled participants and is still ongoing. We present results from the first 6 months of iterative development of Project C, through September 13, 2020.

All procedures in the Brain Safe trial, which was registered at ClinicalTrials.gov (NCT04121858), were approved by the Indiana University Institutional Review Board (IRB). Participants consented to the study, including receiving the app, app-related interactions, and usage data collection, and completed usability and technology acceptance questionnaires. Additional procedures for Project C, for example, providing remote technical support or collecting additional feedback on usability, were conducted for the purpose of improving operations and quality; they were, therefore, not deemed human subjects research. However, changes to allow remote consenting processes with newly enrolled participants were approved as protocol amendments by the IRB.

### Conceptual Model

**Figure 1:**
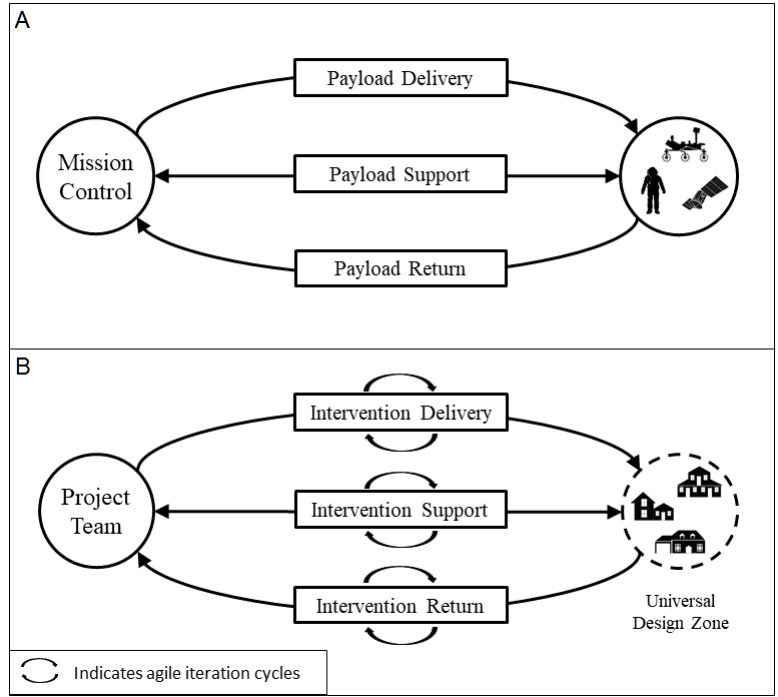
(A) General model of remote spaceflight missions; (B) Framework for Agile and Remote Operations (FAR Ops).

We framed the shift to remote operations as a challenge akin to, but less extreme than, spaceflight. Spaceflight missions send people and payloads (eg, satellites, probes, and rovers) to extremely distant locations, including Mars and the International Space Station [32-34]. Mission success requires solving three challenges: (1) delivery (ie, getting payload to its destination), (2) support (ie, remotely identifying and solving problems throughout the mission), and (3) return (ie, retrieving materials or information during or after the mission) (see Figure 1, A) [35-38]. Similarly, the challenge of remote technology intervention research is three-fold: (1) delivery of the technology to a diverse set of distant recipients; (2) support of the technology and its users, including training, technical support, and communication; and (3) return of data (eg, usability assessments), equipment, and feedback regarding status (see Figure 1, B).

To this spaceflight operations model, we added an Agile project management approach [39] to address the need for innovation and timeliness; the Agile approach is represented by feedback loops in Figure 1, B. The Agile approach was introduced by software developers to promote iterative design, delivering products on a short timeline, responding to user feedback, and learning from failures [40]. Agile approaches have since been used to improve health care delivery, for example, to identify, adapt, implement, and evaluate evidence-based clinical services [41]. Holden and Boustani [42] argued that replacing status quo health care operations with an Agile Mindset could help organizations more nimbly and effectively cope with crises, including the COVID-19 pandemic. In our work, we adopted the three principles of an Agile Mindset proposed by those authors:

1. Sprints. Accelerate progress toward a minimum viable product, to be progressively tested and improved in sprints.
2. Sensors. Embed sensors to collect timely, nonjudgmental, and actionable feedback, leading to learning and redesign between sprints.
3. Safe culture. Practice a culture of psychological safety, such that teams have latitude, decision authority, and resources and, above all, feel psychologically safe to innovate, experiment, and fail.

### Setting and Participants

The study took place in central Indiana, USA, in partnership with Indiana University, Purdue University, Regenstrief Institute, and local not-for-profit academically affiliated health systems. Participants were consenting cognitively normal adults aged 60 years or older, English-speaking, and using prescribed anticholinergic medications; cognitive normality was determined using the six-item cognitive screener [43]. The first cohort of participants, including all described here, were receiving primary care at Indiana University Health, a statewide academic health system. Individuals were excluded if they were diagnosed with, or using medications to treat, dementia or serious mental illness; were living in an extended care facility or not managing their own medications; were unable to use an app due to sensory disability; or screened positive for cognitive dysfunction or terminal illness. All had consented to the Brain Safe trial and provided a waiver of HIPAA authorization.

### Procedure

We applied the Framework for Agile and Remote Operations (FAR Ops) model to achieve technology delivery, support, and return functions in an Agile manner. A team of three study personnel formed the core Project C team: a part-time postdoctoral fellow, with a PhD in industrial and systems engineering; a part-time user experience designer and developer, with an MS in human-computer interaction; and a full-time research specialist, with a BS in neuroscience. The study principal investigator (PI) provided the team with minimal critical specifications to deliver and assess the technology. The team was expected to communicate with the PI, solicit feedback from the broader research team, and develop their own timeline, budget, and procedures. They were explicitly encouraged to be innovative and embrace failure as an opportunity to learn, but to fail early by conducting rapid cycles of testing and redesign, as described in other work on mHealth [44-46].

The team met formally once a week, maintaining informal communications on an as-needed basis, to brainstorm ideas, develop a weekly plan, and monitor the progress of solution development. They worked autonomously and communicated progress to receive feedback and support, not direction, from peers and leaders. The team determined that the first minimum viable product would be a *care package* containing all the necessary resources and information for participants to receive and use the intervention, along with a word search activity book to show goodwill and increase engagement; this token item was approved by the IRB. In the Results section, we further describe various iterations of the care package product, its delivery, and how users were supported postdelivery.

After the first minimum viable product was ready, the team followed a 2-week sprint schedule. For 1 week they remotely delivered a new batch of packages, and in the subsequent week the team collected feedback, analyzed the results, and redesigned the approach for the next week’s deliveries.

To assess technology usability, the team (1) used the Simplified System Usability Scale (SUS) for Cognitively Impaired and Older Adults [47] to obtain a high-level summary usability score and (2) developed a novel usability questionnaire to obtain feedback on specific usability issues with the apps. The novel questionnaire asked for participants’ general impressions of the app (eg, if they had trouble reading the text and if they were happy the app was available) as well as their impressions regarding each step of app use (opening the app, logging in, adding and removing medications, etc). Participants were also given the opportunity to express their opinions in their own words in free-text boxes. Participants were given access to an online version of the questionnaire and/or a paper version to be mailed back to the study team. The questionnaires were one way the team was able to return information from remote participants. Another method to return nonstudy data from users, deployed after the first 6 months and, therefore, not reported here, was a user and activity dashboard, with functionality to create or edit accounts remotely, monitor every log-in and in-app function use, and generate custom usage reports.

### Analysis

We evaluated the performance of the project on the following criteria, as derived from Agile project management metrics:

1. Time to develop and deliver the first package (ie, minimum viable product); our goal was ≤6 weeks.
2. Adherence to the 2-week sprint cycle; our goal was 100%.
3. Percentage of returning usability questionnaires; no goal was set.

This approach is not textbook Agile. Instead, we have adopted a more general Agile-inspired mindset emphasizing quick progress, autonomous work, and a safe culture within the team. We decided against spending a considerable amount of time implementing more Agile metrics in favor of a quicker response. The three metrics listed above were chosen due to their simplicity and their effectiveness in demonstrating whether our approach was successful in terms of time to respond to the challenge, maintenance of an Agile Mindset, and collection of feedback.

We also analyzed and report feedback obtained from participants and team personnel to evaluate the feasibility of remote operations and the Agile approach. An in-depth analysis of collected usability results was beyond the scope of this paper.

## Results

### Overview

The 49 participants who were enrolled in the Brain Safe study but had not yet been delivered a study app, were the target of Project C. The demographics of this target group are included in Table 1. No participants had significant cognitive impairments (eg, dementia).

Three main types of care packages were developed and delivered to participants; Types A, B, and C are summarized in Table 2 with the cost of each. Table 3 details the contents and methods used for each package type. Package types, and iterations within type (ie, A_0_ vs A_1_), were developed between sprints as new needs arose. For example, as some participants began receiving email instructions to download the app to their own smartphone device, the project team learned of the need to provide additional remote installation support.

**Table 1:** Project C (COVID) target group demographics.

Demographic		Number of participants (N=49), n (%)
**Sex**		
	Male	15 (31)
	Female	34 (69)
**Age (years)**		
	60-64	29 (59)
	65-69	8 (16)
	70-74	8 (16)
	75-79	2 (4)
	80-84	1 (2)
	85+	1 (2)
**Race**		
	White or Caucasian	33 (67)
	Black or African American	13 (27)
	More than one race	3 (6)
**Education level**		
	Some high school	3 (6)
	High school graduate or General Educational Development	8 (16)
	Some college	14 (29)
	College degree	14 (29)
	Master’s or other advanced degree	9 (18)
	Other or no data	1 (2)

**Table 2:** Care package types.

Type	Description	Participant needs addressed	Purpose of the package	Cost per package (US $)
A_0_	Full package (version 1)	For participants without their own smartphone or preferring to use a study phone	To fulfil the technical (ie, software, hardware, and internet) needs of participants, some of whom were using a mobile app or modern smartphone for the first time	$34.70
A_1_	Full package (version 2)	For participants who needed fast and direct delivery	Same as A_0_ and to improve the feasibility and lower delivery costs	$17.70
B	Interest-renewal package	For enrolled participants who were difficult to reach in other ways (ie, phone and email)	To re-engage participants who may have lost contact or interest during the pandemic but could not be seen in person	$2.00
C	Remote technical support package	For participants using their own smartphone device and experiencing technical difficulties installing or using the app	To support or troubleshoot self-installation and use of the app	$2.55

**Table 3:** Care package contents and methods.

Function served and package contents and method		Package type			
		A_0_	A_1_	B	C
**Delivery**					
	Postal delivery: delivered via United States Postal Service	✔^a^			
	Hand delivery: driven to participant’s home and left on doorstep by research team member		✔	✔	✔
	Study phone: Samsung Galaxy S9 smartphone with charger and protective case	✔	✔		
	Getting started guide: documentation with study ID, research and support team contact information, and instructions on first-time phone and app use	✔	✔		
**Support**					
	Technical support contact: IT support phone number added into study phone contacts	✔	✔		
	Help documentation: link to online help documentation for the app loaded onto study phone; if requested, printed copy of help documentation was included	✔	✔		
	Video walk-through: link to tutorial video with app usage guidance loaded onto study phone; also accessible within the app’s help section		✔		
	Study phone screen sharing software: Samsung Knox technology management installed on study phones for remote screen sharing and control, with permission	✔	✔		
	Other screen sharing software: Zoom or TeamViewer used for remote screen sharing and control, with permission, for participants not using study phones				✔
**Return**					
	Paper System Usability Scale (SUS): hard copy of the SUS	✔	✔		✔
	Digital SUS: link to online version of the SUS installed on study phones	✔	✔		
	Paper usability questionnaire: hard copy of in-depth usability questionnaire	✔	✔		✔
	Digital usability questionnaire: link to online version of in-depth usability questionnaire installed on study phones	✔	✔		
	Return envelope: prestamped, preaddressed return envelope to return completed paper questionnaires	✔	✔		✔
**Other (ie, goodwill and engagement)**					
	Token item: word search puzzle book and pencil	✔	✔	✔	✔
	Appreciation letter: letter of thanks and explanation from research team to participants		✔	✔	✔

^a^Check marks indicate contents were included in package type.

### Delivery

#### Overview

In the beginning of the pandemic, we faced the challenge of providing mobile apps to older adults who (1) were not tech savvy enough to download and learn to use the app on their own and/or (2) did not possess the technology required to use the app. To address these challenges, two specific conditions related to intervention delivery needed to be met:

1. Smartphones needed to be delivered to those who did not have their own.
2. Participants needed to begin using the study app and smartphone.

#### Smartphone Delivery

Our need to deliver a physical item (ie, a smartphone) to participants who needed one was the primary reason our team created individual care packages for participants. We initially shipped the packages to participants using the United States Postal Service (USPS). We predicted this would eliminate contact between participants and study personnel, in compliance with institutional pandemic restrictions.

Shipping packages with USPS proved efficient in reducing the time required from Project C team members, but left many delivery aspects outside the control of the study team or inconvenient for participants. Due to the contents of the packages being of high value, the mail recipient’s signature was required by USPS. This not only meant that participants needed to be present at the time of delivery, but it also required face-to-face contact between the participant and the postal worker. Each time a participant could not sign for delivery added at least one day’s delay. Shipping the packages also affected the cost per package and the time between package creation and delivery.

In the next iteration of the package, the study team decided to *hand-deliver care packages* to participants, thus reducing sign-off failures, costs, and delays. This also led to the team priming participants by confirming their availability to receive the package and their correct address. Project C staff took appropriate disinfecting, distancing, and personal protective equipment precautions when preparing and hand-delivering packages.

#### Initiating and Sustaining App Use

Prepandemic protocols had study personnel demonstrate how to use the app to participants during an in-person meeting. To familiarize participants with the operation of the smartphone and the steps required to initiate their use of the app, a *getting started guide* was included in the package. Participants were contacted using their preferred method (ie, email, phone call, or text, as specified during their baseline meeting with the study team).

In addition to package components to facilitate use of the app, we included a *token item* (ie, a word search puzzle book) and *letter of appreciation* from the team to show goodwill in difficult times and add something fun to the package. Due to delays between initial recruitment and app delivery as well as stress that was likely being experienced by many participants at the time, we believed these would promote participant engagement with the team and the study.

We experienced difficulties contacting a subset of participants who had agreed to the study in 2019 but had not yet received the app. In an attempt to reach and re-engage those participants, smaller packages containing only the token item and appreciation letter were sent to 2 such participants. This was discontinued in later sprints in favor of more cost- and time-effective methods of reaching out to nonresponsive participants.

### Support

We understood that many participants would require some form of technical support to effectively use the app. We brainstormed many ideas for remote support and over time attempted and refined several. First, we included *contact information for study personnel* (ie, dedicated technical support email and phone number) in each package. We provided in-app one-tap links and a web link to *digital copies of help documentation* demonstrating the features of the app and how to access each app function. Paper help documents were provided as needed.

In later iterations of the package, we created a *video walk-through and tutorial* of the app with voice-over instructions. We determined that seeing the app used on a screen would help some participants learn how to use the app without needing synchronous interactions with study personnel, thus allowing them to use the app at their own pace.

We anticipated that while phone interactions and video tutorials were adequate to address the needs of some participants, there would be others who would benefit from more detailed, personalized support. We implemented *screen sharing capabilities* to offer such support. Study-provided phones were equipped with the option to view and control the phone screen remotely using Knox Manage Remote Support, which was conducted with explicit permission from the participant when needed. This helped when participants could not effectively explain what was on their screen via a phone call and, thus, was viewed as an “invaluable” feature by study personnel.

Additionally, some participants used their own smartphones and were able to download and install the app following email instructions, but required additional remote support. Initially we attempted to use Zoom, a software platform for web-based meetings that allows for screen sharing. We found that installing, registering for, accessing, and using Zoom was too cumbersome for first-time users or caused problems due to system requirements. The team thereafter tested the use of TeamViewer, a similar platform that permits screen sharing by having a participant read a unique 10-digit “Partner ID” to a study team member, leading to on-screen commands to set up and initiate screen sharing. Although TeamViewer required a paid subscription to control the partner’s device, the screen sharing function significantly reduced setup time from over one hour (ie, the time it took for the one attempt to use Zoom) to an average of approximately 10 minutes.

Most privacy concerns about screen sharing were mitigated by openly communicating and building rapport with participants. The screen sharing tools were explained to each participant as well as each step involved in allowing the team member to view their screen. Participants were given opportunities to discuss anything about which they were unsure and it was made clear they could opt out of screen sharing and have technical support delivered solely over the phone instead.

### Return

The simplest viable method was to provide participants with a one-tap link to *digital usability assessments* installed on the study phone. Online assessments ensured uniform delivery and collection of responses in one location. However, we hypothesized that some participants would not want to complete questionnaires on a phone. Moreover, for those not using a study phone, we initially considered but rejected options such as mailing a printed Quick Response (QR) code to the assessment link or emailing the link. Therefore, we added *paper usability assessments* to care packages along with a stamped, self-addressed *return envelope*. We theorized that older adult participants would vary in comfort and ability to complete online questionnaires, but all would be able to return mailed questionnaires.

To increase the return of assessments, the team contacted participants by phone and email, when available. In total, 4 participants remained unreachable after multiple attempts.

### Evaluation

The first of four packages were sent to participants on April 16, 2020, approximately one month from Project C initiation and within the 6-weeks-or-less target. Subsequent deliveries were maintained on the 2-week schedule through June 2020 per our goal; after a delay, package delivery resumed on the 2-week schedule in late July 2020 (see Table 4). The delay was judged as probably unavoidable but constituted a failure to achieve the target pace.

**Table 4:** Care package delivery timeline.

Delivery date	Care package type	Number of packages delivered in batch	Within approximately 2 weeks of prior batch?
April 16, 2020	A_0_	4	N/A^a^
April 30, 2020	A_1_	2	Yes
April 30, 2020	B	2	Yes
May 14, 2020	C	4	Yes
June 4, 2020	A_1_	2	Yes
July 23, 2020	A_1_	1	No
August 5, 2020	A_1_	6	Yes

^a^N/A: not applicable; this was the first delivery date.

During the 6-month period, 19 participants were invited to complete usability assessments in their care packages. Of these, 10 (53%) returned the short, 10-item SUS. Of those 10 participants, 6 (60%) used the link on the study phone, 2 (20%) were emailed the link after providing an email address, and 2 (20%) completed the questionnaire by phone after receiving a follow-up phone call. None successfully returned a mailed SUS, though we do not know how many attempted without success, for example, if their response was lost in the mail due to campus reorganization of mail delivery services. Out of 19 participants, 8 (42%) returned the longer, in-depth questionnaire to the study team. Of those, 5 (63%) used the in-phone link, 2 (25%) completed by phone, and 1 (13%) both returned a paper version and completed the questionnaire online.

Providing email links or having participants respond to assessment questionnaires over the phone are examples of our iterative process enabling our team to respond and adjust our approach based on the unsolicited feedback of our participants. We did not initially have email addresses for our participants and we anticipated that the use of USPS and paper questionnaires would be utilized more frequently than they were. When participants requested that they be allowed to fill out the questionnaire through email, and provided the team an email address, or indicated they had time to discuss the questionnaire and provide answers over the phone, we were able to adjust to meet their needs. While these changes did not constitute a new care package iteration, they are still agile adjustments that our team was able to make as we gained more information about the preferences of our participants.

## Discussion

### Principal Findings

During the COVID-19 pandemic, some clinical trials were forced to shut down [48], while others found ways to adapt [49]. To adapt technology-based research, we created the FAR Ops framework, based on spaceflight operations and Agile processes. Guided by the framework, we iteratively developed and tested solutions, which enabled us to deliver a study intervention and collect usability feedback from older adults, remotely. We succeeded in designing and deploying a minimum viable product in less than 6 weeks, generally maintained a 2-week sprint cycle, and achieved a 40% to 50% return rate for usability assessment instruments.

Our work demonstrates the feasibility of “going remote” to deliver and assess technology interventions to a diverse population during a pandemic and likely in future, less dire circumstances. Researchers and practitioners can replicate our approach as an alternative to face-to-face interactions. Doing so not only permits work to continue during circumstances such as communicable disease outbreaks and natural disasters, but can also save costs, accommodate people with disability or other reasons for staying at home (eg, caregiving responsibilities), reduce travel time and reliance on transportation for participants and staff, allow for testing in natural settings, and overall increase the diversity of participants [11,12,18,50].

The FAR Ops conceptual model provides a framework for such endeavors. Its Agile project management approach can be replicated to ensure goals are met on time, progress is made at a rapid pace, and innovation is embedded in the process by continually refining the solution based on emerging needs and feedback from testing [40]. We demonstrated how simple Agile concepts, such as explicitly setting expectations for a rapid first “good enough” iteration or minimum viable product and short sprint cycles, resulted in rapid, continuous progress.

Our Agile approach also helped the team iteratively respond to new information, which led to adaptations in package design to meet needs, solve problems, and improve effectiveness and efficiency. This is not possible in a traditional or *waterfall* approach to project management, wherein solution development is front-loaded and prolonged in an effort to come up with the ideal solution, leaving little time, opportunity, and resources available to redesign and retest the solution if it turns out to not be as ideal as assumed. In fact, it has been documented that technologies developed without integrating iterative testing and redesign opportunities result in products that do not effectively respond to user needs [51-53]. Compared to waterfall approaches, Agile allows feedback from customers—in our case, participants—to dictate changes, rather than assuming the design team can predict customer experiences [54]. However, because an Agile approach promotes quickly creating the minimum viable product, this can result in early solutions having fewer features. This was true in our case, as participants in earlier iterations did not receive tutorial videos or screen sharing, leading to challenges. At the same time, these challenges inspired these subsequent features to be added, reinforcing the importance of the Agile approach for innovation and determining the contents of the solution based on actual experimentation [55].

We adopted an Agile Mindset, meaning we went beyond specific Agile techniques and even downplayed the need to adopt specific Agile techniques, such as measuring velocity and throughput, diagramming, or having formal scrum teams [56,57]. We emphasized instead the Agile Mindset principle of establishing a psychologically safe culture within which the team was empowered to work autonomously, make quick progress, prefer “good enough” to “perfect,” and learn from failures. However, we acknowledge by some standards, the project’s specific methods were discordant with textbook Agile approaches.

Much like user-centered design, our approach was iterative, catered to diverse participant needs, and made changes to designs based on evidence provided by users [51-53]. We assert that user-centered design and the Agile approach are complementary—the Agile approach can help operationalize user-centered design best practices, while user-centered design compels solutions that fit the end user. The concept of the Agile approach and user-centered design compatibility is detailed in Holden and Boustani [58] and Holden et al [59].

A significant challenge overcome by our study team was the lack of existing methodologies and guidelines available for remote usability testing with older adults. Recently, McLaughlin et al [8] outlined various tools available for different kinds of remote usability testing of medical devices and recommended that study personnel be available for phone support during tests with older adults. Beyond this advice, there is no clear guidance from human-computer interaction, usability, mHealth, or other literature on how to operationalize a fully remote study protocol for older adult participants. Based on the results of our work, we present additional recommendations for remote operations with diverse populations in Table 5.

**Table 5:** Recommendations for remote usability studies with diverse populations.

Recommendation	Details
Screen sharing and screen control	Valuable for technical support Use alongside phone-based support Allows research personnel to more clearly understand participant challenges Use screen sharing platforms that minimize input required from participant (eg, Knox Manage Remote Support and TeamViewer)
Phone-based interactions	Some participants may prefer to be contacted by telephone Allows for participants to talk through impressions and responses to usability evaluations Can enable researchers to gather more detailed responses to open-ended questions Determine if participant would prefer to answer questionnaires over the phone
Email	Some participants may be very familiar with email interactions while others are not Determine if participant has a valid email and would prefer to be emailed a link to a usability questionnaire, over other methods

About 50% of participants returned their usability assessments, fewer than desired and, thus, limiting the success of the project. This may be due to reduced levels of engagement overall, especially during unusual circumstances (eg, the COVID-19 pandemic), or as a result of remote and often asynchronous communication. Prior work recommends one-on-one, synchronous conversation as the most effective method to elicit information from older adults [27,28] and may explain why some participants preferred to complete assessments by phone, even though this option was not initially offered. Synchronous interactions may have been preferred by participants to provide lengthier or supplemental feedback to the team. Indeed, phone responses were, on average, longer than those through self-administered modes. We further hypothesize that some participants may have had difficulty typing their responses or using online questionnaires, which contributed to nonresponse or preference to complete assessments by phone, though this was not communicated to the study team by any participants. This may have been especially so for the longer questionnaire, although response rates for the shorter structured 10-item SUS (53%) and the longer questionnaire with free-text responses (42%) were not exceptionally different. It is important to note that while this flexibility in data collection methodology enabled us to collect more responses from participants, it also introduced variability in the level of detail of collected responses. This could challenge the fidelity of studies that heavily rely on consistent data collection techniques.

Maintaining participant engagement was difficult for our team and may indicate that it would be valuable if, in the future, we were more closely able to replicate face-to-face interactions when assessing intervention usability. We are currently exploring adapting existing synchronous remote usability methods for older adults.

The ages of the 19 participants who received Type A and C packages were, overall, representative of the larger target group: 12 in their 60s, 5 in their 70s, and 1 participant in their 80s. Younger participants were more likely than older participants to return usability assessments to the study team; of those older participants who did return assessments, they were more likely to prefer phone interactions over electronic responses. The two SUS assessments performed over the phone were collected from participants in their 70s, while those in their 60s returned the surveys electronically. Only 1 individual in their 70s completed the longer usability questionnaire, while the rest were returned by younger participants. Although any conclusions are speculative given the small sample size, it may be easier to “go remote” with younger older adults.

A limitation of this study is that it was not designed to compare our approach with others, prohibiting conclusions about its superiority. We also did not design our study to compare various package iterations to each other. During the project, decisions on design iterations were based on relatively small samples of participant feedback and impressions of study personnel. We report only 6 months of the project. Our sample was relatively diverse, but overrepresented younger, White, and female individuals. We believe that limitations with our sample are reasonable due to the nature of the COVID-19 pandemic. We did not formally assess the team’s fidelity to Agile principles or use certain Agile formalisms, such as velocity or control charting, to track progress.

### Conclusions

Combining a spaceflight model with an Agile approach allowed our team to adopt remote operations for our mHealth trial, in response to interruptions from the COVID-19 pandemic. Our approach can be useful for other research or practical projects under similar circumstances or to improve efficiency, cost, effectiveness, and participant diversity in general. Indeed, although the COVID-19 pandemic was the impetus for “going remote,” we believe further iterating remote operations for older adults will result in these approaches being preferable to traditional ones for many reasons.

In addition to offering a replicable approach, this paper tells the often-untold story of practical challenges faced by mHealth projects and practical strategies used, as another article in this journal [45] compels us to do:

Telling and learning from the typically untold stories will result in more efficient, effective, and sustainable mHealth design efforts...We call on our fellow researchers, designers, and UCD [user-centered design] experts to document and share their own challenges and strategies toward improving the implementation of UCD.

## Abbreviations

FAR Ops: Framework for Agile and Remote Operations
HIPAA: Health Insurance Portability and Accountability Act
IRB: Institutional Review Board
mHealth: mobile health
NIH: National Institutes of Health
PI: principal investigator
Project C: Project COVID
QR: Quick Response
SUS: System Usability Scale
UCD: user-centered design
USPS: United States Postal Service
